# Implementation of MOVE UP and Effect on Outcomes

**DOI:** 10.1093/geroni/igaa057.3082

**Published:** 2020-12-16

**Authors:** Kieffer Lori, Linda Semler, Jennifer King, Brandi Boak, Elizabeth Venditti, Anne Newman, Steven Albert

**Affiliations:** University of Pittsburgh, Pittsburgh, Pennsylvania, United States

## Abstract

The MOVE UP behavioral activation program, consisting of 32 sessions over 12 months, was delivered by trained community health workers (CHWs) at 26 sites. 300 participants completed a mean of 21.5 sessions. Change in body weight was associated with site attendance: among 9 sites with mean attendance < 70%, participants lost a mean of 5.3%; among 12 sites with 70-80% attendance, 5.6%; and among 5 sites with > 80% attendance, 9.2%. Completion of activity and diet logs followed a similar pattern (34.9%, 56.2%, and 72.7%, respectively), as did retention for 13-month outcome assessment (70%, 85%, and 88%, respectively). CHWs at the high-performing sites were more likely to have prior or current employment in weight management and fitness (90% vs. 41.7% and 44.4%), but did not differ in education, age, race, or employment by sites. CHW experience, not sociodemographics, affected outcomes.

